# Molecular and Physiological Characterization of Two Novel Multirepeat β-Thymosins from Silkworm, *Bombyx mori*

**DOI:** 10.1371/journal.pone.0140182

**Published:** 2015-10-16

**Authors:** Shangshang Ma, Zhiqiong Kang, Peng Lü, Yanhua Yang, Qin Yao, Hengchuan Xia, Keping Chen

**Affiliations:** Institute of Life Sciences, Jiangsu University, Zhenjiang, Jiangsu, P. R. China; Institute of Plant Physiology and Ecology, CHINA

## Abstract

β-thymosin plays important roles in the development of the lymphatic system and the central nervous system in vertebrates. However, its role and function in invertebrates remain much less explored. Here, we firstly isolated a gene encoding β-thymosin in silkworm (*Bombyx mori* L.). Interestingly, this gene encodes two polypeptides, named as BmTHY1 and BmTHY2, via two different modes of RNA splicing. The recombinant proteins fused with an N-term GST tag were over-expressed in *Escherichia coli* (*E*. *coli*) and further purified to near homogenity to prepare mouse antibodies. The Western blot analysis showed that these proteins were expressed in various tissues and organs, as well as in different developmental stages. Amazingly, the expression of BmTHY2 was hugely increased during the pupae stage, indicating a specialized role in this period. The expression of these proteins was gradually decreased in BmN cells infected by BmNPV, suggesting they may play different roles in the virus infection. In addition, both BmTHY1 and BmTHY2 can interact with 14-3-3 of silkworm and Ubiquitin of BmNPV as shown by GST pull down and Co-IP assays, consistent with their roles in the regulation of the development of nervous system.

## Introduction

Thymosin, a group of small peptides originally extracted from calf thymus, was firstly found by Goldstein et al in 1966 [1]. Based on the different isoelectric point of the extracted component, it can be classified into three types: α-thymosin (pH < 5.0), β-thymosin (pH 5.0~7.0), γ-thymosin (pH > 7.0) [2, 3]. Currently, most studies have focused on β-thymosins, and Tβ4 (Thymosin β4) is the most intensively studied in vertebrates. Tβ4 is a major actin sequestering proteins, which can efficiently prevent F-actin assembly [4]. By interacting with ATP synthase, it facilitates itself binding to G-actin and assists nucleotide switch [5, 6]. It has been shown to play an important role in the lymphatic system development and immune system maintenance [7]. In addition, it can promote wound healing [8], cell migration [9, 10], cardiac repair [9], and regulate central nervous system [11].

Up to date, most studies on β-thymosins have been carried out in vertebrates, and there are only a few studies performed in invertebrates. Even so, it is becoming increasingly clear that these two kinds of β-thymosins behave very differently. In vertebrates, β-thymosin usually contain only one thymosin domain (THY) that can bind to actin and exists as an active monomer. In contrast, multiple copies of THY domains are often found in invertebrate β-thymosins, which are called multirepeat β-thymosins [12]. In addition, almost every species have several β-thymosin isoforms in lower eukaryotes and protists, and similarities between them are very high [13]. Moreover, these isoforms are often encoded by the same gene [14, 15], suggesting that they may have similar functions [16, 17]. Recently, multirepeat β-thymosins have also been shown to be involved in the immune response [7] and promoting cell migration, reducing ROS [15], promoting the development of molting animals [14], participating in nervous system development and tissue [16, 18], organ regeneration [19].

A recent study suggested that the multirepeat β-thymosins in cotton bollworm may participate in molting and antivirus response [14]. By contrast to cotton bollworm, silkworm is a beneficial insects of significant economic interests, which is susceptible to the BmNPV infection. To investigate the physiological and immunological roles of β-thymosins in silkworm, we cloned their encoding gene. It encodes two β-thymosins by alternative splicing. We expressed them in *E*. *coli* and further purified for antibody preparation and protein-protein interaction experiments. We found that both proteins can interact with actin and 14-3-3 proteins, consistent with their roles in the regulation of actin networks and development of nervous system. They are expressed widely in various tissues, organs and developmental stages. Notably, the BmTHY2 is greatly up-regulated in the pupae samples, indicating it may have a specialized role in this stage. However, unlike the situation in cotton bollworm, the expression of these proteins were gradually decreased in BmN cells infected by BmNPV, suggesting they may play different roles in the virus infection process.

## Materials and Methods

### Materials

The *Bombyx mori* strain 306 [20], BmN cell [21]were maintained in our lab, gastric cancer cells SCG-7901 was a gift from Professor Shi(Bogoo Lot: BG463, China). Silkworms were reared on mulberry leaves under standard conditions. The midgut, testis, ovary, head, fatty body, hemolymph from the fifth instar larvae were collected, frozen immediately in liquid nitrogen, and stored at -80°C. Nascent eggs, first-fifth instar larvae, pupae (3 days after pupation), and moths were also frozen in liquid nitrogen and stored at -80°C. Hemolymph-derived BmNPV BVs were purified according to the method of Chen et al [22].

### Bioinformatics Analysis

The Sequence were aligned using Mega 5.0. The Genedoc server was then used to shade identical and similar amino acid residues black and grey, respectively (60% conservation).

### Cloning of BmTHY1, BmTHY2

The BmN cDNA was used as template to amplify BmTHY1 and BmTHY2 ORF by PCR using following primers. F: 5’-CGGGATCCCC ATGGCCTGCTCCGTGAGTGAC-3’; R: 5’-CCCTCGAG TCAAGCTGATTTCTCTTGCTC-3’. The underlined are *Bam* H I and *Xho* I recognition sites. The PCR products were purified using the kit (Sangon Biotech code: GK2043-50, China). After digestion with *Bam* H I and *Xho* I, the purified PCR products were subcloned into the expression vector pGEX-5X-3, using T4 DNA ligase (Takara Code: D2040, Japan). And the positive colonies were identified by enzymatic digestion and PCR. The constructs pGEX-BmTHY1 and pGEX-BmTHY2 were verified by DNA sequencing (Sangon Biotech, China).

The genomic DNA was extracted from the midgut of a silkworm (Sangon Biotech code: SK8221, China). The introns were identified by PCR with primers: Genomic-F (5'-TTGTTTGTTGTTTATAGATTCACAATGGCCTGC-3') and Genomic-R (5'-TAACTGTTATAAAGTAGTGGTTCAAGCT-3'). Actin A3: F (5'-ATTTACTAAGGTGTGCTCGAACAGTGCGC-3') and R (5'-CTGTTGGCCTTGGGGTTCAGGGGAG-3'). The PCR products (6000 bp) were verified by DNA sequencing (Sangon Biotech, China).

### Protein Expression, Purification, and Mass Spectrometry

The recombinant plasmid was transformed into *E*. *coli* BL21 (DE3) competent cells, which were incubated at 37°C in liquid LB culture media containing 50 mg/mL ampicillin. The expression of the GST fusion protein was induced at an A600 of 0.6 with a final concentration of 1 mM IPTG (isopropylthio-β-Dgalactoside). The glutathione S-transferase (GST) Resin chromatography (TransGene Biotech code: DP201, China) was used to purify the recombinant proteins BmTHY1 and BmTHY2, as instructed by the manufacturer manual. The concentrated proteins were digested by Factor Xa (BioLabs Lot: 09212211, Germany) and further purified. Solution was removed by dialysis. The 12% SDS-PAGE was performed to determine its molecular weight and analyzed by MS System (ultraflex-TOF-TOF).

### Western Blot

Polyclonal antibody was prepared by immunizing Kunming mouse (Laboratory Animal Research Center) using purified BmTHY2 as antigen. 100 μg of BmTHY2 (equal to about 1 mL of the antigen/adjuvant mix) was injected into the abdominal cavity of a mouse. In total, 4 times of immunizations were done at one-week intervals. During the third week, the serum of mouse tail blood was used to detect the efficiency of antibody. Serum was collected 7 days after the last boost, and then stored at -20°C. The experiments were performed with formal approval from the Animal Ethics Committee of Jiangsu University. The animals were handled in accordance with the Guide for the Care and Use of Laboratory Animals of the National Institutes of Health.

The total protein extracts from BmN cells, silkworm different tissues or samples of different development stages were prepared as described by Lü et al [21]. Pierce the tail then collect the hemolymph. The protein concentration was determined by the Bio-Rad DC Protein Assay method (Thermo Fisher Scientific Lot: KI138546, USA). Protein samples were equalized and the electrophoresis was carried out using 12% SDS-PAGE, and proteins were transferred to polyvinylidene difluoride (PVDF) membranes with constant current of 200 mA for 35 min. The membranes were blocked with 5% skim milk in TBST (pH7.5), incubated with anti-BmTHY IgG as the primary antibody. Then, the membranes were washed and incubated with secondary antibody anti-mouse IgG (Sigma, China). Membranes were washed three times with TBST and TMB reagent (Seajet Scientific Inc, CAS-No: 54827-17-7, China) was applied to visualize protein bands. Every SDS-PAGE and Western blot were carried out at least three times.

### Protein Binding Assays *In Vitro*

The GST pull down was performed according to Luo et al [23]. Briefly, the GST fusion protein GST-BmTHY1 or GST-BmTHY2 was loaded onto Glutathione Sepharose beads, then 100 μg of the total protein of silkworm midgut (infected with or without BmNPV) was added and incubated for four hours. After extensive washing step to remove unbound proteins, the bound proteins were eluted using the elution buffer containing 10 mM reduced glutathione, and then subjected to SDS-PAGE and Western blot analysis. The GST proteins loaded onto Glutathione Sepharose resin were used as negative control.

The co-immunoprecipitation was performed as described in the manual (Pierce® (Co-IP) kit (Thermo Corporation, Item 26149, USA). Similarly as above, the silkworm midgut proteins were used to detect the target protein. The anti-β-Actin mAb (Vazyme Biotech Co. Lot: Ab101-01/02/03) and the anti-14-3-3(Santa Cruz, Lot: sc-1020) were for coupling resin.

### Cell Proliferation and Migration Assay

The MTT method was used to determine the effect of BmTHY1 and BmTHY2 on the growth of gastric cancer cells. The proteins were mixed with a DEME medium to a final concentration of 0.1 or 1 mg/mL to culture gastric cancer cells in 96-well plates, and non-treated cells were used as a control group [15]. 12 hours later, cell proliferation was determined by MTT kit (Number: C0009, Beyotime Biotechnology Co, Ltd.).

To determine whether these proteins effect on cell migration, the gastric cancer cells were cultured in six-well plates, and a pipette nozzle (4 * 49 mm) was used to draw a line to generate an empty space for new cells to migrate into [24]. The line would serve as fiducial marks for the wound areas to be analyzed. In preparation for making the wound, the free serum medium was used to prevent cell growth. Similar amount of proteins were used as mentioned above. 12 hours later, the cells were observed under a microscope (Leica). Images were analyzed by digitally drawing lines (using Adobe Photoshop) averaging the position of the migrating cells at the wound edges. The cell migration distance was determined by measuring the width of the wound divided by two and by subtracting this value from the initial half-width of the wound [25]. Data were analyzed using Statistical Package for the Social Sciences version 18.0 (SPSS) software to examine the biological significance with the Student’s t-test analysis.

## Results

### Bioinformatic Analysis of β-Thymosins in *Bombyx mori*

**Fig 1:**
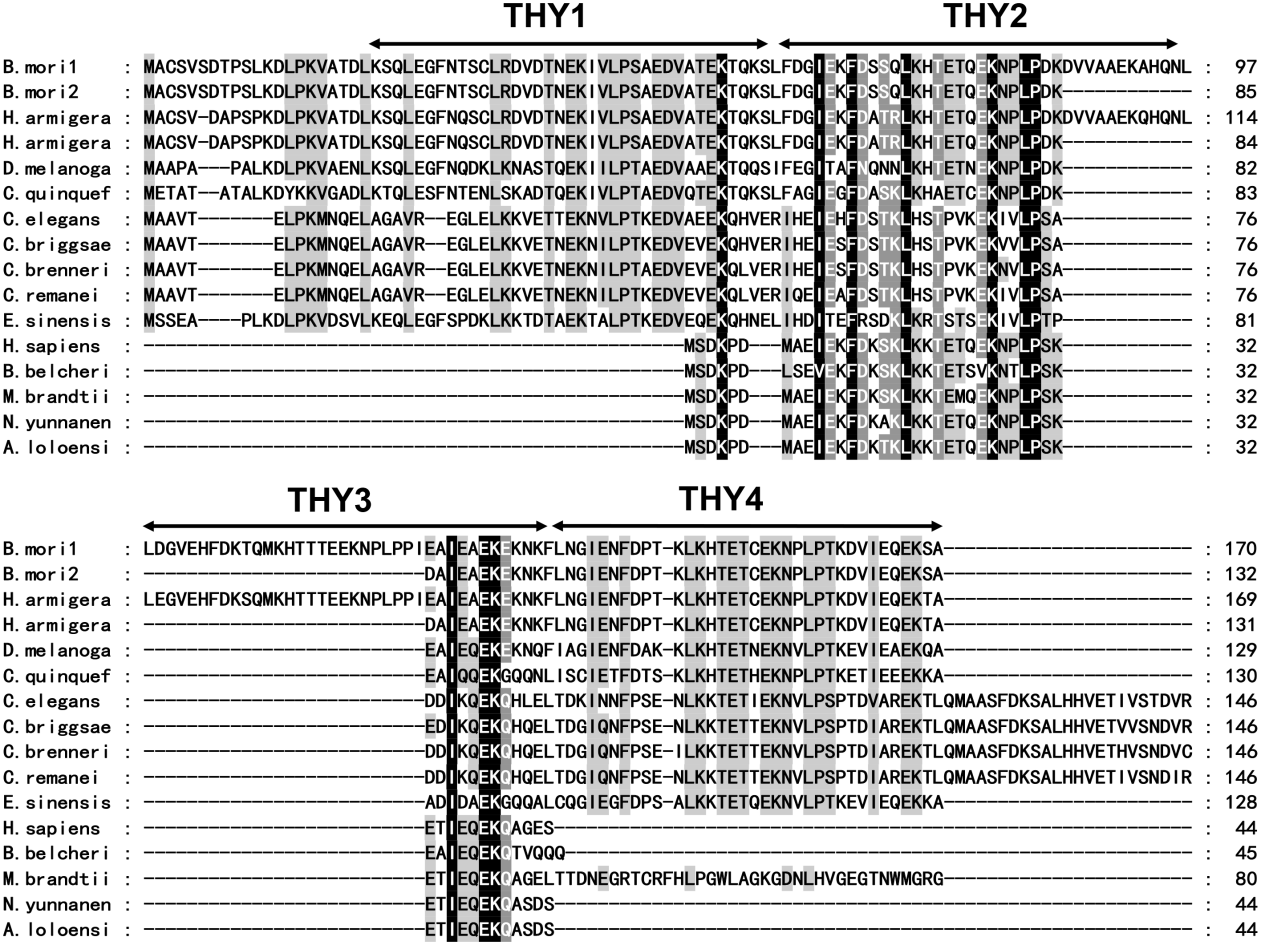
Sequence alignment of BmTHYs. Amino acids are colored according to the conservation (Black represents the most conservative, followed by dark gray, then the weakest is light gray) GenBank numbers are as follows: *Bombyx mori* thymosin isoform 1,2 (NP_001040486.1, NP_001103818.1), *Helicoverpa armigera* thymosin isoform 1,2 (ADD21556.1, ADD21557.10), *Culex quinquefasciatus* (XP_001863288.1), *Drosophila melanogaster* (NP_525065.1), *Caenorhabditis elegans* (NP_509430.1), *Eriocheir sinensis* thymosin-repeated protein 1 (ACP19740.1), *Homo sapiens* (NP_066932.1), *Branchiostoma belcheri* (AAK72482.1), *Myotis brandtii* (EPQ12153.1) *Nanorana yunnanensis* (ABQ12776.1), *Amolops loloensis* (ABG78789.1).

We amplified two specific bands using PCR method, named as *BmTHY1* and *BmTHY2*. As revealed by sequencing and BLAST analysis, the coding regions of *BmTHY2* are completely included in that of *BmTHY1*. Compared to protein sequences from other species, the two cloned BmTHYs protein sequences show a high identity with them, including HaTHYs from *H*. *armigera*, Cib B from *D*. *melanogaster* etc. Notably, only the second and the fourth THY domains are highly similar to vertebrate THY, the first THY domain is quite different with binding sites mutated to "LRDV" from" LKK/HT", and the third one is unique that exists only in the lepidoptera insects (Fig 1), suggesting the lepidoptera insects including silkworm and cotton bollworm may have specific roles for such an organization of β-thymosins. The binding motif of each THY domain for G-actin is quite different from "LKHT", which is very conservative in vertebrate. Besides, the silkworm β-thymosins also contain an elongated N-terminal sequence like other invertebrate β-thymosins, which is shown to be able to enhance the affinity of binding to G-actin compared to vertebrate β-thymosins [13].

**Fig 2:**
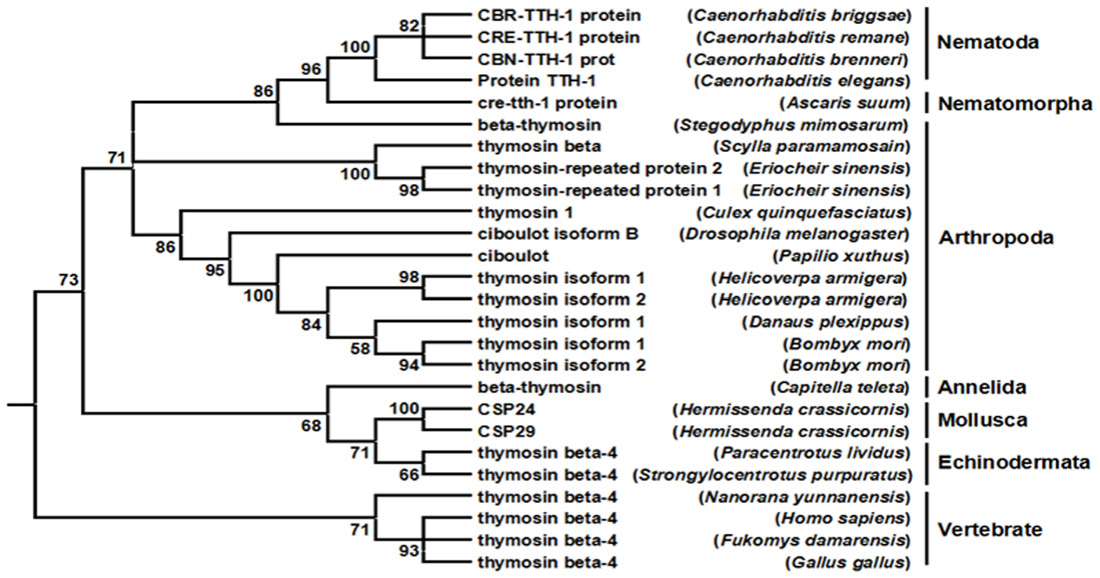
Phylogenetic analysis of BmTHYs. Phylogenetic tree of various β-thymosins. The tree has been arbitrarily rooted between a clade consisting of the monomeric forms in vertebrate. GenBank numbers are as follows: *Caenorhabditis briggsae* (XP_002645512.1), *Caenorhabditis remane* (XP_003100241.1), *Caenorhabditis brenneri* (EGT34446.1), *Caenorhabditis elegans* (NP_509430.1), *Ascaris suum* (ERG81070.1), *Stegodyphus mimosarum* (KFM79832.1), *Scylla paramamosain* (ACY66642.1), *Eriocheir sinensis* thymosin-repeated protein 1,2 (ACP19740.1, ACP19741.1), *Culex quinquefasciatus* (XP_001863288.1), *Drosophila melanogaster* (NP_525065.1), *Papilio xuthus* (BAM17917.1), *Helicoverpa armigera* thymosin isoform 1,2 (ADD21556.1, ADD21557.10), *Danaus plexippus* (EHJ77183.1), *Bombyx mori* thymosin isoform 1,2 (NP_001040486.1, NP_001103818.1), *Capitella teleta* (ELU01379.1), *Hermissenda crassicornis* CSP24, CSP29 (AAN08024.1, AAN08022.1), *Paracentrotus lividus* (CAD29144.1), *Strongylocentrotus purpuratus* (NP_999791.1), *Nanorana yunnanensis* (ABQ12776.1), *Homo sapiens* (NP_066932.1), *Fukomys damarensis* (KFO19279.1), *Gallus gallus* (NP_001001315.1).

As shown by the following phylogenetic tree analysis (Fig 2), it is clear that multirepeat β-thymosin is not a synapomorphy of the ecdysozoa, it also exist in annelida and mollusca. Note that, the monomeric in echinodermata and the multirepeat form in annelida and mollusca are in a clade, suggesting that the two forms are orthologous, which is incompatible with previous study [26]. Since other ecdysozoa phyla are not documented, Our phylogenetic tree is consistent with the previous study based on 18S rRNA sequence of β-thymosin which suggested that arthropoda be closely related to nematode worms [27].

### The Genomic Sequence of BmTHYs

**Fig 3:**
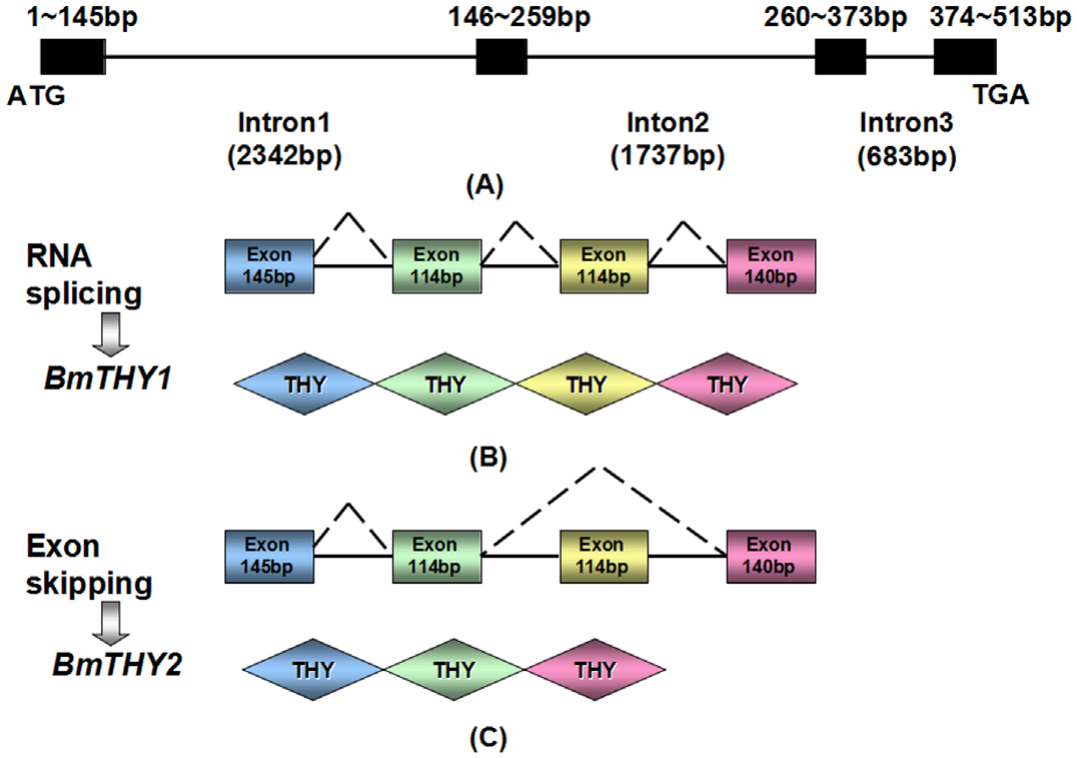
Genomic structure and organization of BmTHYs. The top panel shows the genomic organization (A), the middle (B) and bottom (C) panels show the splicing patterns for BmTHY1 and BmTHY2, respectively. The introns are shown as lines and exons as boxes. Splicing sites are indicated by the diagonal dashed lines.

To examine whether the two cloned cDNAs from BmTHYs are derived from the same gene, we cloned the corresponding genomic DNA sequence. By Genomic-F and Genomic-R, it could generate two bands using BmN cDNA as a template, while there exist only one product using the genomic as a template. Sequencing (S2 Fig) analysis revealed that this gene contained four exons: exon 1 (145bp), exon 2 (114bp), exon 3 (114bp) and exon 4 (140bp). *BmTHY1* contains all the exon sequences, while *BmTHY2* only includes exon1, 2 and 4 (Fig 3).

### Expression, Purification of Recombinant BmTHYs

**Fig 4:**
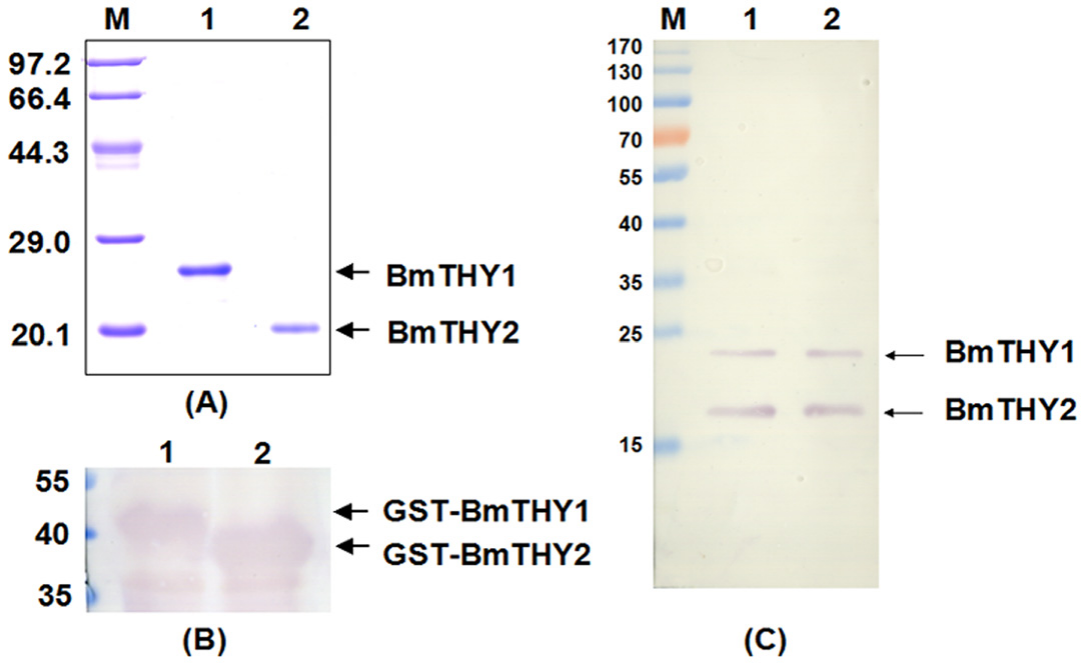
The expression, purification and Western blot analysis of recombinant BmTHYs. The expressed BmTHYs proteins were purified, digested to remove GST tags and further purified and subjected to 12%SDS-PAGE (A). The prepared antiserum was used to Western blot to detect the BmTHY1 and BmTHY2 tagged with GST from the lysate of *E*. *coli* expressing the corresponding proteins (B), the BmN cell lysate (C, lane 1) and the protein extract of ovaries (C, lane 2). (A)Lane M, protein molecular weight marker; Lane 1: BmTHY1; Lane 2: BmTHY2. (B)Lane 1: GST-BmTHY1; Lane 2: GST-BmTHY2. (C)Lane M, prestained protein ladder; Lane 1: The total protein of BmN cells; Lane 2: The total protein of ovaries.

Recombinant BmTHYs were expressed in *E*. *coli* and purified by GST affinity Chromatography (Fig 4A). As expected, the molecular weight of BmTHY1 is about 22 kDa and that of BmTHY2 is about 19 kDa, and they were expressed correctly(S1 Fig). The mouse polyclonal antiserum were prepared and successfully used to detect the GST-BmTHY1 and GST-BmTHY2, as shown by the Western blot (Fig 4B). And it is clear that this antibody could be used to investigate the protein expression profiles for BmTHYs (Fig 4C).

### Expression of BmTHYs in Different Tissues and Various Silkworm Developmental Stages

**Fig 5:**
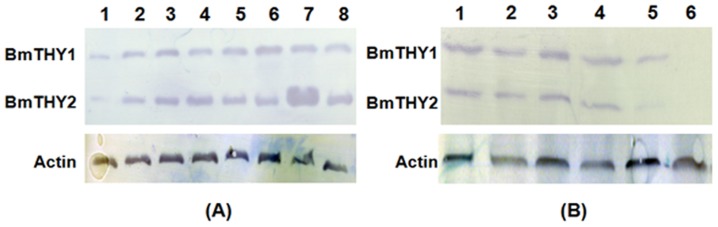
The Spatiotemporal expression profiles of BmTHYs. Western blot analysis of the expression levels of BmTHYs in different developmental stage (A) and in different tissues and organs (B). The mass of each lane’s total protein were 50 μg, and the sample were equalized. (A) Lane 1: egg; Lane 2: 1st instar; Lane 3: 2nd instar; Lane 4: 3rd instar; Lane 5: 4th instar; Lane 6: 5th instar; Lane 7: pupae; Lane 8: moth. (B) Lane 1: midgut; Lane 2: testis; Lane 3: ovary; Lane 4: head; Lane 5: fat body; Lane 6: hemolymph.

Western blot was performed to determine BmTHYs expression levels. As shown in Fig 5A, the two β-thymosins were widely expressed in all the samples covering the whole life cycle of silkworms. Amazingly, during the pupae stage the BmTHY2 were greatly over-expressed, indicating it may be involved in metamorphosis.

On the other hand, to elucidate the distribution of BmTHYs, we examined its expression in different tissues and organs, including the midgut, testis, ovary, head, fat body, and hemolymph of fifth-instar larvae. In Fig 5B, both proteins exist in all samples except the hemolymph, and the BmTHY2 is clearly down-regulated in fat body compared to BmTHY1. Together, these data indicate the BmTHY2 may have more complex regulatory incidents compared to BmTHY1.

### Interactions of BmTHYs with Ubiquitin of BmNPV and 14-3-3 of Silkworm

**Fig 6:**
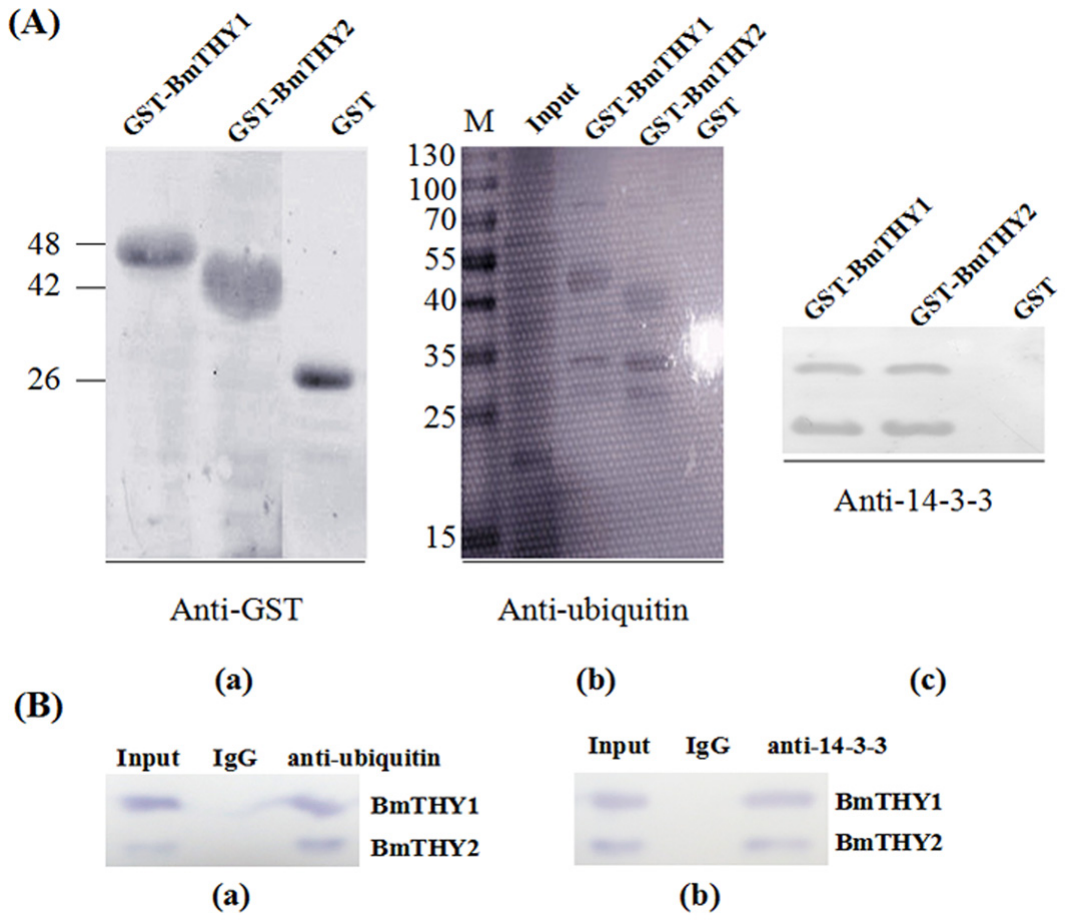
The protein-protein interaction analysis. The total protein of midgut was used for the GST pull down (A, B) and Co-IP (C, D) to detect proteins interacting with BmTHYs. The GST tagged BmTHY1 and BmTHY2 were purified and used to pull down actin (A) and 14-3-3 (B). The Co-IP was performed using anti-actin (C) and anti-14-3-3(D) antibodies as baits, and anti-BmTHY2 antiserum was used for Western blot analysis.

To explore which protein of midgut can interact with BmTHYs, GST pull down and Co-IP assay were carried out. The results showed that both of them can bind to 14-3-3. Unexpectedly, using the infected midgut as samples, we found both of BmTHYs can interact with Ubiquitin of BmNPV (Fig 6).

### Expression Pattern of BmTHYs in BmN Cell Infected with BmNPV

**Fig 7:**
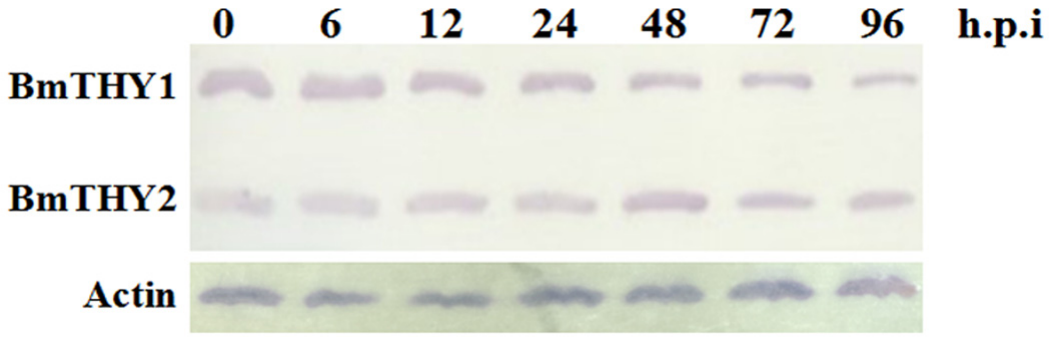
The expression pattern of BmTHYs in BmN cells infected with BmNPV. The BmN cells were infected with BmNPV and samples were collected at different hours to examine the expression of BmTHYs. The mass of each lane’s total protein were 30 μg, and the sample were equalized.

After BmN cell was infected with BmNPV (BV), the BmTHY1 expression decreased gradually (Fig 7) and was significantly decreased starting from the 24 hours after the infection, indicating it may play directly antivirus role.

### The Effect of BmTHYs on the Gastric Cancer Cell Proliferation and Migration

**Fig 8:**
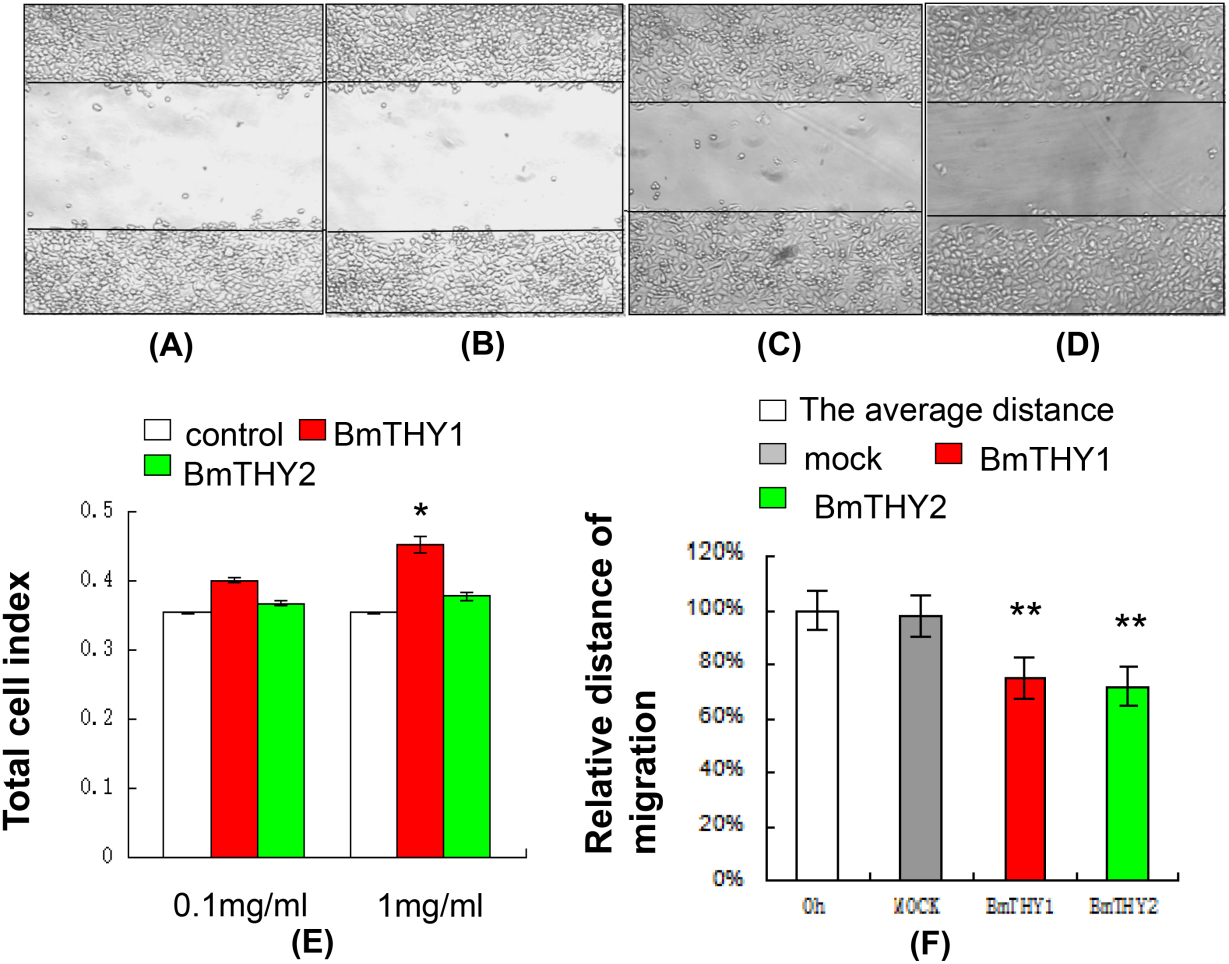
The effects BmTHYs on cell proliferation and migration. A: The initial status after scratch. B: 12 hours later, cell grew, the distance narrowed (without any reagents). C: 12 hours later, BmTHY1 efficiently promote cell migrate. D: 12 hours later, BmTHY2 efficiently promote cell migrate. E: MTT assay to detect the effect of BmTHYs on cell proliferation, * *P* < 0.05. F: Analysis the effect of BmTHYs on cell proliferation, ** *P* < 0.01.

The gastric cancer cells have been used in previous studies to examine the effect of thymosins on cell proliferation and migration, and thus were also employed in this study. As shown in Fig 8, both BmTHY1 and BmTHY2 could promote the proliferation of gastric cancer cells, and BmTHY1 seemed to have a stronger effect than BmTHY2. In addition, they both could effectively stimulate the migration of gastric cancer cells.

## Discussion

To investigate the roles of β-thymosins in the silkworm development and immunity against pathogens, we cloned the encoding genes, *BmTHY1* and Bm*THY2*. The *BmTHY2* sequence is identical to the β-thymosins gene *BmTHY* reported recently. However, they did not identify the *BmTHY1* gene [28]. It may be caused by the different cDNA samples used as PCR templates. It is clear that the expression of BmTHY2 but not the BmTHY1 is extremely increased during the pupae stage, as revealed in our data (Fig 5A). Thus, the abundance of BmTHY1 in their cDNA samples is much lower compared to that of BmTHY2, rendering it hard to be detected by the PCR. Fortunately, we used cDNAs prepared from BmN cells where both genes showed similar expression levels, thus both genes had similar opportunity to be amplified, which was also supported by our Western blot analysis (Fig 5A). Therefore, to our knowledge, we for the first time had identified complete genes for silkworm β-thymosins.

As revealed by the genomic sequence analysis, the BmTHY1 containing 4 exons may be produced by constitutive splicing (Fig 3), and the BmTHY2 containing 3 exons may be produced by alternative splicing (Fig 3). Interestingly, the two transcripts (or cDNAs) show high similarity to those in cotton bollworm, suggesting similar splicing patterns may exists in lepidoptera insects. The different splicing mechanism could generate multiple isoforms of β-thymosins, and it could obviously increase the complexity of the organization of THY domains to fulfill various roles in invertebrates. Indeed, the EsTRP2 can promote the proliferation of human hepatocellular carcinoma cell, but the EsTRP1 cannot [12].

During the silkworm development process, the two β-thymosins are continually expressed, indicating a requirement for their functions. Amazingly, the expression of BmTHY2, but not the BmTHY1, is hugely up-regulated in the pupal stage, indicating a specific role of BmTHY2 in this period (Fig 5A), which is also consistent with the behavior of β-thymosins in cotton bollworms [14]. On the other hand, both BmTHYs are expressed in most tissues and organs tested except the hemolymph, for they lack secretion signals consistent with findings in Tβ4 [8]. Compared with the previous paper (2012) [28], we got some different results, this may be due to the different use of silkworm strains. Although the transcripts could be detected in hemolymph in our experiments (S3 Fig), they could not be observed by Western blot.

As shown in Fig 6, both BmTHY1 and BmTHY2 can interact with silkworm 14-3-3 proteins (Bm14-3-3ζ and Bm14-3-3ε) [29]. These proteins are universal adapters participating in multiple cellular processes. Previous studies in *Hermissenda crassicornis* suggested that the post-translational modifications of Csp24 (a β-thymsoin protein) regulate its interaction with 14-3-3 and contribute to the enhanced cellular excitability of the nerve system [18, 30]. Indeed, during the soldier differentiation in *Hodotermopsis sjostedti*, the expression of both 14-3-3ζ and β-thymsoin are very high [31, 32]. Thus, it is possible that BmTHYs may participate in the development of nervous system formation of silkworm.

It has been demonstrated that nuclear filamentous actin (F-actin) is required for nucleopolyhedrovirus (NPV) progeny production in NPV-infected lepidopteran cells [33]. Many components are involved in nuclear F-actin assembly, such as G-actin, actin-related protein 2/3 complex 2/3 (Arp 2/3), and an N-WASP homologous protein from various viruses. These N-WASP proteins can activate Arp2/3 to initiate the F-actin polymerization, and their homologues have been shown to be P78/83 of *Autographa californica* multiple nucleopolyhedrovirus (AcMNPV) [34], as well as HA2 of *Helicoverpa armigera* nucleopolyhedrovirus (HearNPV) [35]. Interestingly, β-thymosins are also homologous to N-WASP proteins, suggesting these silkworm β-thymosins may also be involved in the actin network regulation during the NPV infection process. Indeed, β-thymosins have been reported to be important immune-related factors and involved in progresses of anti-viral and anti-inflammatory [7, 14, 36, 37]. However, by contrast to the up-regulated transcription of homologous genes in cotton bollworm induced by virus infection, the protein levels of BmTHYs appeared to be decreased during the course of BmNPV infection (Fig 7), indicating it may be degraded and may play different roles in the virus infection of silkworms. Though both BmTHYs can iteract with Ubiquitin of BmNPV, it is clearly the two were not degradated by ubiquitination (Fig 7). In human, Tβ4 can be degraded by angiotensin converting enzyme (ACE) [38], and we found the homologue of this enzyme in silkworm, so we speculated that silkworm thymosin can also be degraded by ACE, but the physiological role for this interaction still unclear.

Currently there are still many debates on the roles of Tβ4 in the regulation of cell proliferation. It was reported that the mRNA encoding Tβ4 was increased rapidly in tumor cells and the overexpression of Tβ4 could promote the cell grow and migrate [39]. However, application of β-thymosin could not repair the damaged skin of animals [40], and the β-thymosin could turn tumor cells into normal cells [41], indicating it has no or even inhibitory effects on cell proliferation. In this study, the BmTHY1 seems to have stronger effects to stimulate cell proliferation than BmTHY2 (Fig 8), although both have similar stimulating effects on cell migration (Fig 8), which is consistent with Tβ4’s role in the regulation of G-actin polymerization and depolymerization [9, 10].

Up to date, most studies on β-thymosins have been carried out in vertebrates, and there are only a few studies performed in invertebrates. On the basis of the existing research, we found that the role of thymosin in each species has a certain similarity. There are many different kinds of thymosin isomers in invertebrates, and the distribution of different species is not exactly the same, different isomers play a role in mutual compensation, these function be completely the same, or may be entirely opposite.

## Supporting Information

S1 Fig The result of Mass spectrometry. (TIF)

S2 Fig The genomic sequences of BmTHY. (PDF)

S3 Fig Cloning of BmTHYs using different tissues as templates. (TIF)
