# Supplementary figures and images for: Molecular and Physiological Characterization of Two Novel Multirepeat β-Thymosins from Silkworm, Bombyx mori
Source: PLoS One. 2015 Oct 16;10(10):e0140182. doi: 10.1371/journal.pone.0140182 (PMC4608725; doi:10.1371/journal.pone.0140182)

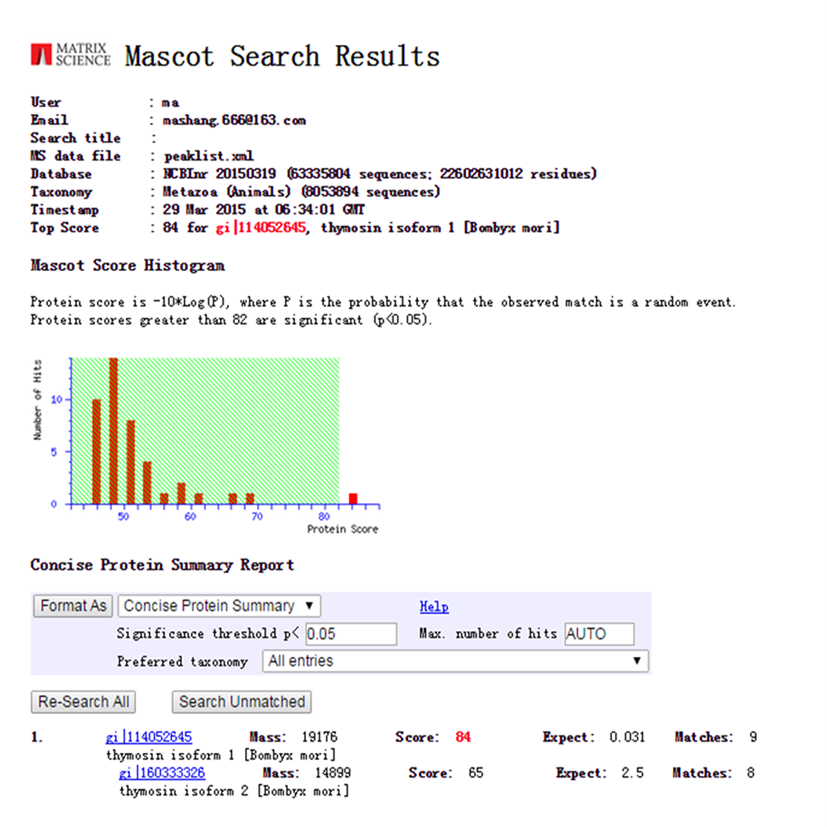

Supplement: S1 Fig — (TIF) [file pone.0140182.s001.tif]

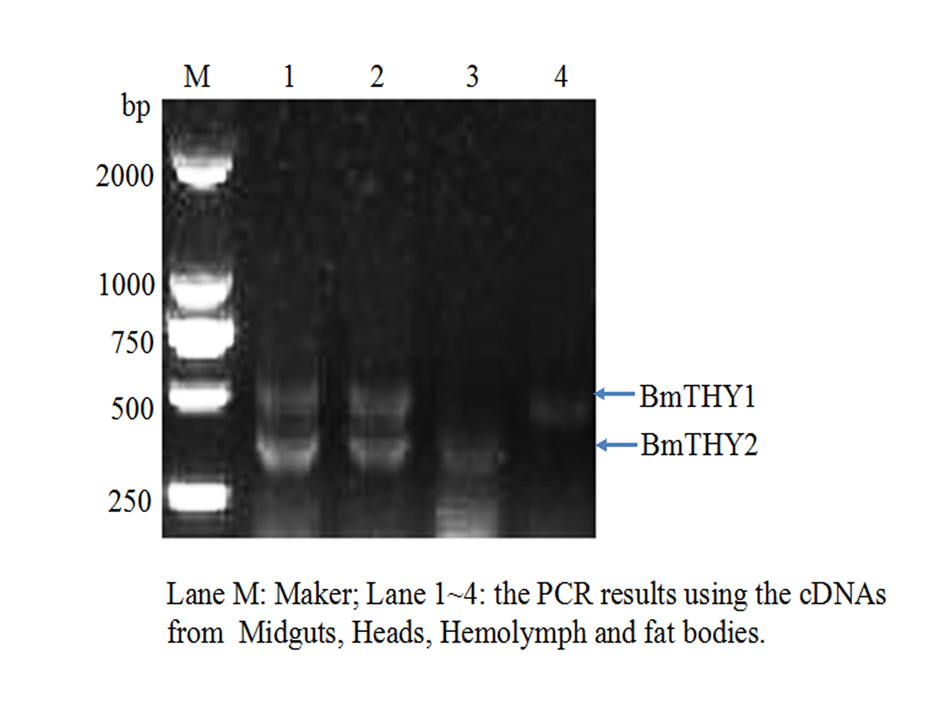

Supplement: S3 Fig — (TIF) [file pone.0140182.s003.tif]
