# Supplementary material for: Molecular and Physiological Characterization of Two Novel Multirepeat β-Thymosins from Silkworm, Bombyx mori
Source: PLoS One. 2015 Oct 16;10(10):e0140182. doi: 10.1371/journal.pone.0140182 (PMC4608725; doi:10.1371/journal.pone.0140182)
Supplement: S2 Fig — (PDF) [file pone.0140182.s002.pdf]

TTGTTTGTTGTTTATAGATTCACA**ATGGCCTGCTCCGTGAGTGACACTCCCTCCCTGAAAGACCTCCCCA**
AGGTCGCCACAGACCTGAAGAGTCAGCTCGAAGGCTTCAACACCAGCTGTCTCCGTGACGTCGACACCAA
TGAAAAGATTGTGCTTCCGTCTGCTGAAGGTAGGAATAAGAGTACCCTGCATATGTTTTTCACGCGAAAT
CTGAAATACCTATTCACACCTAAGTTCTATAGATATATATTAAGATTATTTTTGGTTTTTTAATTACAGT
TCTCGCGCGTACACTTGGAATATAGCTCCGAGTTCAAACTCAAATTAAACTAAATTAAATAAAAAAATAA
AAATGAAATTAAAGTTATCATACGACAGTCAAATATAATAGCTAATAAAATTAAGTATACCTATAAAATA
TTACAGCCAATAAAATTATATTACAGCTAAAAGGAACACCACAAACAAGAACCATCGAAAGAGATTTTTT
ACTTTATCTATTTTTTTTTTTAATTCCACCACAGACGTGGCAAAAGAGAAGCAACACAACGCTTTACTAC
AAGGCGTTGAACTATTCCAACCGTCTTCATTAAGGAAAACCGAGACAATAGAAAAAAACATTCTCCCTAA
CGCAATAGGTATTCATTCGCTGGGTTGAAATAACAACTAACGCGAAGACGCCGTGTACTTACTTACATGA
GCTTTGTTTTATTTCGTTGTCCAATAGACAACTTTTTTAACATATTTAATCAAGCGTAAGATTTATCTTC
TGTACACTAAGAATGCTTAAAGTCATCAATAATTTAAACTATTACTGTGCACATACGAAATAACTCTGCC
ATTCTGAACAATCCATACTTCACGCATGTGTTGTGAATAGTTTTAATGTAGATCTATGCGGATTTTAAGT
AAATATTTAATCGAGTGATGTCATTATTATTAACTAGCGGCCCGTATCGGCTCCGCTCGGGTTTTTAACA
AAAATTTCAACGATATTTGACCTTGTTTTATTTTTTTAAATAAAAGAACACTTATTGCGGCATAACTATA
ATAGTTAGTTATATGCTGTCGCGACGCTTTTTGTAAATAATAATGCGTTCTACAAAGTCGTAGTATATTA
TTTTATTCTATCATCAATAGTTTTCGCAGGGCACGCGATGTAAAGAATATTTTAGGTAATTTTTTACACC
TTGGGTTACATTATTGGAGTTTTAGTAAGGATCCCAAATTTTTTTCAAAAAAGATTATAGCCTATGTCAC
TCGAGAATAGTGTAGTTTCCAAACAGTGAAAGAATTTTTCAAATCGGTTCAGTAGTTTCGGAGCCTATTC
AATACAAACAAACTAACAAATCTTTCCTCTTTGTAATATTAGTATAGATATCAACATGCTTCGACGCGCG
CGACCACTTGCGCGGATTTAACGCTGTCAAATCAGTCCGCGAAAAATTACTGAAAGCACGCATTTGGCGC
TATATAGAAAACTTTCTAGAACCATAAACTTTCGTTTTTAAACGTAAACTTCAAACTTGTTTATTTTACT
CTGAACTCCAAGAACTAGCAAAAAACTTTATACTGTTTTTTAAGTCAGCGTTACTATTACATAATAACAA
CATTTGTTTACGCTACGTATTTTATAACAACGCGCATGTTTTGTAAATGTTGTATGTATGTGTGTTTGAT
AAAGAAATAATAACAATTTGAACGGGGGCAAGCAGACGGGCTCGCGGTGAACGGCGCCACCCGTCCTAAA
GAGACGAACATTAAATTATAACGGAACGAAACACAATGGCTTCGAATCTGAACGCATGACTGCTTCTCAA
CATATTCACGATTGACTTACACGATGAAGGAATAACATCGTGTAATAAAAATCAAACCCGCAAAATTATA
ATTTGATAAATACAATTATAGTATACTATACTAATTACTGGTTGTAGGACCTCTTGTGAGACCCCGCGGG
TAGGTACCACCACCCTGCCTATTTCGGCCGTGAAGCAGTAATGCGTTTCGGTTTGAAGGGTGGGGCAGCC
GTTGTAACTATACTTGAGACCTTAGAACTTATATCTCAAGGTGGGTGGCGCATTAACGTTGTAGATGTTT
ATGGGCTCCAGTAACCACTTAACACTTGTGAGCTCGTCCACCCATCTAAGCAATAAAAAAAATATATATA
TGACTAGCTGACCCGGCAGACTTCGCAGTGCCTCAATCGGTAAACAAAAGACCTAAACTTTTGTACAACT
TATAACAAACAAGGGATCCGTCTGACGGGGGACACATCAAAGGAAAAACAAAATTATTATTTTTATTTAA
TTCCGGGCGTTTTCATATTTACATATCTACTTTTTAAACCTTCTCTGGACTTCCACAAATAATTCAAGAC
CAAAATTAGTCAAATCGGTCCAGCCGTTCTCGAGTTTTAGCGAGATTAACGAACAGCAATTCCTTTTTAT
ATATATAGATAAGATGATAGATGAGGTAGTTTTTAATTTTGTTCGTCGTTGATTTTTGCAG**ACGTCGCCA**
CTGAGAAGACCCAGAAGTCTTTATTCGACGGTATCGAGAAGTTTGATTCGAGCCAGCTGAAGCACACCGA
GACTCAGGAGAAGAACCCGCTTCCGGACAAAGACGGTACGTTTGTGTTTCCACAATACTCCGTATATTTG
CTAAACGCGCGCATGCAGTTTGCATTTTGTTATGTTTCAATCATAAACGTTGTGTATGTGCTTTGAATGA
AATGGGAATTGATTTAGAAATACTATGGCTGCGAGCGAGCATTCGTCTTGTAATTAACAGATTCTCGTCA
ATAGAGAGTCCATTGGTCATCGCATTCCGTGACTTGAGCTTAAACAAATCTACAATCGCAATATTAGTGG
ATTAATTATCGTCTAAAAGACGAATCGATTCCCTTTTTGTTTTGAAAGAACGCATACACAGAATATACAT
AGTATTTTACTGTTTGTCTCTTTCTAGTATTTTTAAGATAGTAATTCCGCATATTCGAGTCATCGCAATA
ATAACTAAGGTAATAAGAGAAGCTATCTTTTAGTCTCCACACCTAGTTTTCCCGCAAGGCATGTGTTTAA

ATAGACTGTTTGACTAGCGCGTTTTAACTTAATTAAAGAATTACGCAAATTTGCTTGTATCTTTTAGCTC
AAATAAATATTAAGAAAAACCTGATGTTAAATGATTACCGAAGTCCACAGATCTTCCATAAATGACCCCC
TCACTTTGCAAGATGAGAGCTTAATTTGTTTGAAGCACCCTTTTCCGATCACGTTCTTTGTAATTCATGA
CTAAAGAAAGGTAATTCTAAATTTATAGAAGTGTTTTAATTGCGTTAATTCCTTATTATTATAGTACTCT
TTACGCTCCTAGAAAGAGATGAACAACTAGTTTCGTAGCGTTAGAAATATACTGAAATATGTCTTTATAC
CCTTTCGCTTCACACATTTCTTCACGCTACCAATATTGTCACAAAACATGGTTGTGTTGCACTCTGTACT
TGCAGCAGAATCTTCAGAATTTGACTTGTTTTTGTAGGTCACATTCTTTCTCGTCTCCAAACTTTGTCAA
GGTCATCATCAGACCGACAGTACCATATGTTAGCCGATCTTTTCTTAACCTACCCACACTTATTGATGCG
AGCTTTATGTACCTATTTATATTAAATTAAATACGGGGTTGGCGATCTGAATTTCATCATCAAAGGAACT
ATTCGACAGATGATACATGGCAGTTTTATTAAACTATTTGAGAATATTTTTTGTAGGTTATTTTTTAGTG
AAAACATTGAATATTCTGATTCATTGTTCAATTGATTCGTAAAGTAATAGAGGGAATAATTTCAAACAAA
AGAGCGACTGATTTGTTTAACTCTTGTGTGTCTGTTGTTTATTCGAAACAGCACGATCAGTTTTTAAGAA
TATTTCAACACTAGCGACCCGCCCTCGCTTCGCTTAGGGAACTGTAATTTATTATTGATAACAGTATTTC
TCCACTATTTAATGGATGATATTATACATAAAAACCTTCCTCTTCAATCACTCTTTAAAAAAACCGCATC
AAAATCCGTTGCGTAGTTTTAAAGATTTAAGCATGCCTACATAGGGATACAGGGACAGAGAAAGCGACTT
TGTTTTATACTATGTAGTGATTTATTTATACTATTAGTGATTTATAATGAGACATTATAATATATAATAG
TTAAATGAAGGCGCTATGCTTTATGAAGCTCTTAGAATATTCAGTTGTAGTTTATAAAAACTTATTCAGT
ATCTTTACTCGCCTCGTCGCCATCCCCGGATTCGATTGTTACAATATAATGCCTGCTTTATATGCCGTTT
GCACGATTCTGCTTTGCACCAG**TTGTCGCAGCGGAGAAAGCCCACCAGAACCTCTTGGACGGAGTTGAAC**
ACTTCGACAAGACTCAGATGAAGCACACGACGACGGAAGAAAAGAATCCACTGCCCCCGATCGAAGGTTC
CTTCTTTTATGCTACCGCTTTTGAGCGTTTTAGTTTTTGCACTTTTATAGTCTTTTATAGCTTTTTGTAA
AATGGCATGTTTTAGCTTAACCGTTGCCTTAAGATGATAATAATGAAATCGAGATAATATTCACAGAATC
TGTGTCGTTCAGACTGTTTCCTTCAAAATAGGAACGTTGTAACATTTTGTTAAGAAAAGACATTGAAAAA
AGTTTTTTCAATCTGTTAATTCATTTGAATTATTCAAACTAGATTACAACCGACTTTCTGTCACGAAGTA
ATATCGCGTTCCGCTTTGGAGGTTGACACGACGGTTGTACAACACAATTGAGACTTGACCTCATGTCTCA
AGGAGGATAGAGGATTGAAAAAAAGTCAGATCCAATTAAGTACACACCCGATAAAAAACGTTTGCATCCA
ATCCTATATGTCTGGATCTAGTTATATCTGCTTATATCCAGATACATCCAAATTACTGATCTTCTTGATT
CTTAACATGCACGGGCTGGTACTACTCTCCTAATTACTTTACTCAACTAGGTTGAGTTGGTCCAGTTTGA
AATGTTTTGGTTAATCGCGATCATGGACGATGAATCTGAACCGTGCGTAACGCACAGGGCAGAGGTAACA
ACTTGGGTATTTCGTAAAACAAAATCGTTAATAATTGCTTTCGTCGCAG**CTATCGAAGCGGAGAAGGAAA**
AGAACAAATTCCTGAACGGCATCGAGAACTTCGATCCCACTAAGCTGAAGCACACGGAAACGTGCGAGAA
GAACCCGCTCCCCACAAAGGACGTCATTGAGCAAGAGAAATCAGCTTGAACCACTACTTTATAACAGTTA
CT

Note: The red sections are four exons, while the black parts between the red ones are intones.
